# Hydrological Regime and Water Shortage as Drivers of the Seasonal Incidence of Diarrheal Diseases in a Tropical Montane Environment

**DOI:** 10.1371/journal.pntd.0005195

**Published:** 2016-12-09

**Authors:** Laurie Boithias, Marc Choisy, Noy Souliyaseng, Marine Jourdren, Fabrice Quet, Yves Buisson, Chanthamousone Thammahacksa, Norbert Silvera, Keooudone Latsachack, Oloth Sengtaheuanghoung, Alain Pierret, Emma Rochelle-Newall, Sylvia Becerra, Olivier Ribolzi

**Affiliations:** 1 Géosciences Environnement Toulouse, Université de Toulouse, CNES, CNRS, IRD, UPS, Toulouse, France; 2 MIVEGEC (UMR CNRS-IRD-University of Montpellier), Montpellier, France; 3 Oxford University Clinical Research Unit, Hanoi, Vietnam; 4 Institut de la Francophonie pour la Médecine Tropicale (IFMT), Vientiane, Lao PDR; 5 Irstea Montpellier, Montpellier, France; 6 IRD, Department of Agricultural Land Management (DALaM), Ban Nogviengkham, Xaythany District, Vientiane, Lao PDR; 7 IRD-iEES-Paris, Department of Agricultural Land Management (DALaM), Ban Nogviengkham, Xaythany District, Vientiane, Lao PDR; 8 iEES-Paris (IRD-Sorbonne Universités-UPMC-CNRS-INRA-UDD-UPEC), Université Pierre et Marie Curie (UPMC), 4 place Jussieu, Paris, France; 9 Department of Agricultural Land Management (DALaM), Ban Nogviengkham, Xaythany District, Vientiane, Lao PDR; University of California San Diego School of Medicine, UNITED STATES

## Abstract

**Background:**

The global burden of diarrhea is a leading cause of morbidity and mortality worldwide. In montane areas of South-East Asia such as northern Laos, recent changes in land use have induced increased runoff, soil erosion and in-stream suspended sediment loads, and potential pathogen dissemination. To our knowledge, few studies have related diarrhea incidences to catchment scale hydrological factors such as river discharge, and loads of suspended sediment and of Fecal Indicator Bacteria (FIB) such as *Escherichia coli*, together with sociological factors such as hygiene practices. We hypothesized that climate factors combined with human behavior control diarrhea incidence, either because higher rainfall, leading to higher stream discharges, suspended sediment loads and FIB counts, are associated with higher numbers of reported diarrhea cases during the rainy season, or because water shortage leads to the use of less safe water sources during the dry season. Using *E*. *coli* as a FIB, the objectives of this study were thus (1) to characterize the epidemiological dynamics of diarrhea in Northern Laos, and (2) to identify which hydro-meteorological and sociological risk factors were associated with diarrhea epidemics.

**Methods:**

Considering two unconnected river catchments of 22 and 7,448 km^2^, respectively, we conducted a retrospective time series analysis of meteorological variables (rainfall, air temperature), hydrological variables (discharge, suspended sediments, FIB counts, water temperature), and the number of diarrheal disease cases reported at 6 health centers located in the 5 southern districts of the Luang Prabang Province, Lao PDR. We also examined the socio-demographic factors potentially affecting vulnerability to the effect of the climate factors, such as drinking water sources, hygiene habits, and recreational water exposure.

**Results:**

Using thus a mixed methods approach, we found *E*. *coli* to be present all year long (100–1,000 Most Probable Number or MPN 100 mL^-1^) indicating that fecal contamination is ubiquitous and constant. We found that populations switch their water supply from wells to surface water during drought periods, the latter of which appear to be at higher risk of bacterial contamination than municipal water fountains. We thus found that water shortage in the Luang Prabang area triggers diarrhea peaks during the dry and hot season and that rainfall and aquifer refill ends the epidemic during the wet season. The temporal trends of reported daily diarrhea cases were generally bimodal with hospital admissions peaking in February-March and later in May-July. Annual incidence rates were higher in more densely populated areas and mostly concerned the 0–4 age group and male patients.

**Conclusions:**

We found that anthropogenic drivers, such as hygiene practices, were at least as important as environmental drivers in determining the seasonal pattern of a diarrhea epidemic. For diarrheal disease risk monitoring, discharge or groundwater level can be considered as relevant proxies. These variables should be monitored in the framework of an early warning system provided that a tradeoff is found between the size of the monitored catchment and the frequency of the measurement.

## Author Summary

Aiming to identify the most critical drivers of diarrhea epidemics in montane areas of South-East Asia, we conducted a retrospective time series analysis of diarrhea reported cases and of hydro-meteorological variables measured in two contrasted river basins in northern Laos, together with socio-behavioral practices. We found that during drought periods, the population switches its water supply from wells to surface water where we measured numbers of fecal indicator bacteria (*E*. *coli*) of 100–1,000 MPN 100 mL^-1^ (the World Health Organization recommendation is 0 MPN 100 mL^-1^ in drinking water supply sources). We found that water shortages trigger diarrhea peaks during the dry and hot seasons and that rainfall and subsequent aquifer refill ends the epidemic during the wet season. As *E*. *coli* counts in surface water are high all year long, *E*. *coli* may not be a relevant indicator of the fecal risk in this area. Conversely, stream discharge and aquifer water level appear to be relevant proxies to predict diarrheal risk. Mitigating both environmental and anthropogenic drivers of fecal bacteria dissemination in developing countries is critical in the context of global change: more intense rainfall combined to the extension of run-off prone land uses (e.g. tree plantation without understorey) may decrease both the aquifer recharge and the contaminant retention ecosystem service, and thus threaten the availability of safe water resource for the population.

## Introduction

The global burden of diarrhea is a leading cause of morbidity and mortality worldwide. Acute diarrhea, which is defined as 3 or more loose watery stools within a 24-hour period [1], threatens the linear growth of children [2], increases school absenteeism and contributes to loss of productivity [3]. Despite continuous improvements, diarrhea killed about 1.3 million people globally in 2013, mostly children in developing countries [4]. Diarrhea is most commonly caused by gastrointestinal infections. The primary cause of diarrhea is via the ingestion of infectious agents, especially bacterial or viral, from human or animal feces (fecal-oral route). Modes of transmission include ingestion of contaminated food or water (e.g. through flies, bad sanitation facilities, sewage and water treatment systems, cleaning food with contaminated fluids), direct contact with infected feces, person-to-person contact and poor personal hygiene [5–8].

Microbial contamination is globally widespread and affects all drinking water source types [9,10]. Indeed, in most developing countries, access to improved drinking water sources and adequate sanitation remains a problem despite improvements in recent years [11]. In Lao PDR, as in most developing countries in the tropics, diarrheal diseases are among the leading causes of premature death, ranking 5^th^ in 2013 with about 104,000 years of life lost (YLLs), after lower respiratory infection, neonatal preterm birth, ischemic heart disease and cerebrovascular disease [4]. Young children are still the first victims of diarrhea, which is a major cause of malnutrition and the fifth cause of death under 5 years in 2013 [12]. Over 80% of cases are acute watery diarrhea, usually due to enteric pathogens, especially rotaviruses and *Escherichia coli* (*E*. *coli*) pathovars [13].

Etiological agents of diarrheal diseases (bacteria, viruses, protozoan) naturally occur in aquatic environments in both temperate and tropical areas and the epidemics of the different agents have a specific seasonality [14]. Exploring the seasonality of diarrhea reflects the relative predominance of its etiological agents and can provide new information for future, targeted vaccination programs and scheduled health information campaigns [15]. Further, understanding the determinants of seasonality can help in the development of early warning systems [16].

Meteorological conditions have different effects on the transport, diffusion, reproduction, and persistence of the various pathogens causing diseases such as diarrhea. They also affect human behavior and the timing and intensity of seasonal epidemics. Several authors have investigated the meteorological drivers of the seasonal patterns of diarrhea in both temperate areas [17–19] and tropical areas [16,20–31]. In tropical areas, most studies found that temperature positively correlated with diarrhea epidemics [16,20–22] whereas some other studies found a positive correlation between diarrhea epidemics and relative humidity [16,20,23]. Furthermore, some studies have found a positive association between diarrhea epidemics and rainfall [16,20,24,25] while other studies have shown the opposite [26–28], often related to water shortage and the shift in use towards less safe water sources (e.g. stored water) [29–31].

Diarrhea epidemics were also associated with changes in air pressure [23] and vegetation index [27]. De Magny et al. (2008) showed that chlorophyll A concentration and sea surface temperature were useful predictors of cholera epidemics [32]. River water level was identified as a reliable predictor of diarrhea epidemics [21,33,34]. Diarrhea epidemics have also been related to the El Niño–Southern Oscillation [35–37].

In water bodies (e.g. rivers), fecal indicator bacteria (FIB) are used as a proxy to detect waterborne fecal pathogens at limited cost. The term FIB describes the range of bacteria that inhabit the gastrointestinal tract of homeothermic animals and includes *E*. *coli* and the fecal coliforms, *Enterococcus spp*., all of which are permanently excreted in fecal material [38]. *E*. *coli* was recommended by the World Health Organization (WHO) due to its better performance as an indicator of the pathogens’ presence in water samples, particularly in tropical regions and its stronger association with diarrheal risk [39–41] and other waterborne diseases [42]. Kostyla et al. (2015) found that fecal contamination in drinking water sources in developing countries was higher during the wet season and that this trend was consistent across FIB, water source types, climate zones, and across both rural and urban areas [43]. Several studies have investigated the association between water quality, including FIB counts, and diarrheal disease counts. In their review focused on developing countries, Gundry et al. (2004) highlighted that no clear relationship could be found between point-of-use water quality and general diarrhea incidence [44]. More recently, Kulinkina et al. (2016) found that association between diarrheal risk and microbial contamination in South India was inconsistent except in urban sites where disease risk increased with higher median household total coliforms concentration [25]. Few et al. (2013) found that the environmental contamination in the Mekong Delta river water was high throughout the year and became slightly higher during the dry season, although there was no evidence of elevated diarrhea disease in the dry season opposed to the flood season [45].

Contradictory findings among seasonality studies and the absence of straightforward relationship between water quality and diarrhea incidence highlight the complex relationship between hydro-meteorological and ecological factors and the transmission of waterborne diseases [46]. Additional risk factors (e.g. geographical region, type of water supply, urban vs. rural setting, human behavior, age, host susceptibility, concurrent disease such as HIV) modify this relationship [34,45,47–52]. Mitigating the burden of diarrheal diseases appears especially critical since global change, including climate change, global population increase and urbanization, may impact the ecology of infectious diseases and enhance the outbreak of waterborne diseases [5,53–56], in particular in developing countries where the population is considered to be less resilient than in developed countries [57].

In montane areas in South-East Asia, recent changes in land use (e.g. from subsistence shifting agriculture to cash crops and tree plantations) have increased soil crusting, runoff, soil erosion and in-stream suspended sediment loads [58–60]. Floods and the consequent erosion drive FIB dissemination in surface water in Laos [61–64]. To our knowledge, few studies have yet applied mixed methods approaches to assess diarrheal disease risk factors [45], and no studies have given an estimate of diarrhea incidences, together with water quality factors such as river discharge, suspended sediment and FIB loads, along with the commonly assessed climate factors such as temperature and rainfall, and with sociological factors such as hygiene practices. Also, no studies have yet related diarrhea epidemics to hydro-meteorological drivers at the catchment scale, where rivers can be viewed as an integrator of water, solute, and solid fluxes in a catchment [65]. In this study, we hypothesized that climate factors combined with human behavior would drive diarrhea incidence, either because higher rainfall, leading to higher stream discharge, suspended sediment loads and FIB counts, are associated with higher numbers of reported diarrhea cases during the rainy season, or because water shortage leads to the use of less safe water sources during the dry season. Using *E*. *coli* as a FIB, the objectives of this mixed methods study were thus (1) to characterize the epidemiological dynamics of diarrhea in Northern Laos, and (2) to identify which hydro-meteorological and sociological risk factors were associated to diarrhea epidemics.

## Materials and Methods

### Study design

**Fig 1:**
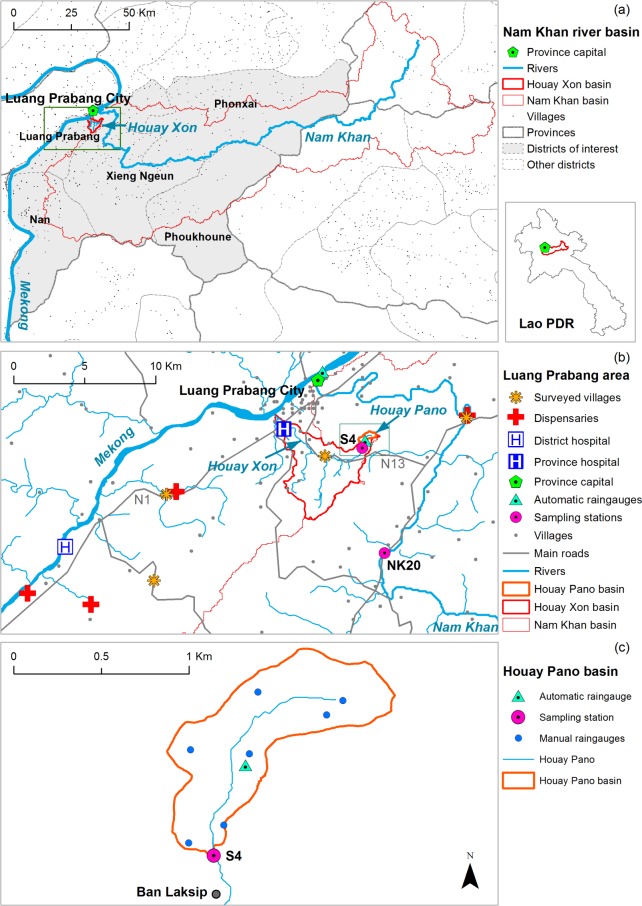
Location of (a) the Nam Khan basin with delineation of the districts of interest in this study, (b) the Luang Prabang area with Nam Khan, Houay Xon and Houay Pano catchments together with sampling stations S4, NK20 and health centers and villages where sociological surveys were performed, and (c) zoom on the Houay Pano catchment with the location of the village of Laksip and meteorological stations.

We conducted a retrospective time series analysis of meteorological variables (rainfall, air temperature), hydrological variables (discharge, suspended sediments, FIB counts, water temperature), and diarrheal diseases visits to 6 health centers located in the 5 southern districts of the Luang Prabang Province, Laos (Fig 1). We also examined sociological factors (pathways) potentially affecting vulnerability to the effect of the hydro-meteorological factors (hazard) on diarrhea incidence (outcome), such as drinking water source, hygiene habits, and recreational water exposure. We thus explored how variations in pathways could affect the relationship between hazard and outcome by combining quantitative and qualitative analyses [45].

### Study area

The study area is located in northern Lao PDR and includes the neighboring watersheds of the Nam Khan River and the Houay Xon River, both located on the left bank of the Mekong river in the Luang Prabang (LP) area (Fig 1). Both rivers are first order tributaries of the Mekong River but are not connected. The Nam Khan River (Fig 1A) flows into the Mekong at Luang Prabang, the only city and major commercial, transport and touristic hub in the area (ca 50,000 inhabitants in 2012). The Houay Xon (HX) River (Fig 1B) connects to the Mekong 4 km downstream from the confluence of the Nam Khan with the Mekong. The Nam Khan and Houay Xon catchments are 7,448 and 22 km^2^, respectively. The Nam Khan is monitored at the NK20 station (Fig 1A). Within the Houay Xon catchment, the Houay Pano (HP) catchment (Fig 1C) is one of its headwater sub-basins (0.6 km^2^). The Houay Pano catchment is monitored at the S4 station.

The climate is tropical sub-humid. It usually can be divided into 3 seasons: a dry and cold season lasting from October to February, a dry and hot season lasting from March to April and a wet and hot season from April to October. The mean annual rainfall at Luang Prabang is 1,268 mm with a coefficient of variation of 28% (1960–2006). In average, about 91% of rainfall occurs between April and October, i.e. during the wet season which we can define as the succession of months with precipitation >80 mm. Annual rainfall over the 3 years of interest in this study (2010–2012) varied widely, with 1,195 mm in 2010 (wet season lasting 6 months from April to September), 2,272 mm in 2011 (wet season lasting 8 months from March to October) and 1,664 mm in 2012 (wet season lasting 8 months from April to November).

More detail about the Nam Khan and the nested Houay Xon and Houay Pano catchments, including the land use and livestock units, have been presented in previous studies [61,63,64,66].River monitoring and sampling allows recording discharge and water quality data integrating the catchment-scale spatial heterogeneity of rainfall, soil and land use upstream the monitoring stations [65]. We used catchments of different sizes also to assess if the dynamics of FIB may be different depending on the catchment scale and if the links between environmental variables and epidemiology may be stronger and more visible at some scales than at others.

### Epidemiological data survey

Retrospective hospital admissions data were collected from January 1, 2010, to December 31, 2012, in the 6 health centers in the Luang Prabang area, Laos: the Province Hospital, the District Hospital and 4 dispensaries (Fig 1B). Data on diarrhea cases were obtained from the declaration forms of the compulsory declaration of the Lao national monitoring system for the notification of infectious diseases (NCLE surveillance system, 17 infectious diseases). We pooled together as diarrhea: acute watery diarrhea, acute diarrhea with blood (dysentery), food poisoning, and typhoid fever. Diarrhea cases were recorded per day. Except for typhoid fever, no information about the causative agent was available: the diagnostic was therefore only symptomatic [67]. Data of daily incidences were analyzed anonymously.

The district and village where each patient was coming from was available in the epidemiological dataset and we summed up the cases for different spatial subsets according to this information. As this study was focused on the Luang Prabang area, we only considered reported cases of patients coming from Luang Prabang city and from the villages included in the 5 districts (Luang Prabang, Nan, Phonxai, Phoukhoune and Xieng Ngeun) overlapping both the Houay Xon / Houay Pano catchments and the downstream area of the Nam Khan basin (Fig 1A, Table 1). We also considered 3 spatial subsets of this dataset, in order (1) to pool together populations as representative as possible of the drainage area upstream sampling stations (S4 and NK20), and (2) to assess the spatial variability of the incidence of diarrheal diseases in the Luang Prabang area. We chose the Xieng Ngeun (XN) district to compare with the Nam Khan basin’s hydrological monitoring at the NK20 station (Fig 1, Table 1) because the major part of the population in the Nam Khan basin is concentrated in the lower third of the basin (75% of the villages and 81% of the population). To compare with the S4 station, we chose two nested spatial subsets: a first subset includes the 7 villages located along the Houay Xon River (Ban Donekang, Ban Khoy, Ban Khuathineung, Ban Laksip, Ban Ma, Ban Phoumork, and Ban Sangkhalok) and a second subset only includes Ban Laksip which is located along the Houay Pano River, downstream the S4 station (Fig 1, Table 1).

**Table 1:** Spatial information used to mix epidemiological, hydro-meteorological and sociological datasets in the Luang Prabang area, Laos.

Epidemiological spatial data aggregation	Hydro-meteorological data	Sociological data
5 districts (Luang Prabang, Nan, Phonxai, Phoukhoune, Xieng Ngeun)	Nam Khan at NK20 and Houay Pano at S4	4 villages in the Luang Prabang area (Donekang, Long Lao, Napho, Xieng Lome)
Xieng Ngeun district	Nam Khan at NK20	
7 villages in Houay Xon valley (Donekang, Khoy, Khuathineung, Laksip, Ma, Phoumork, Sangkhalok)	Houay Pano at S4	
Laksip village		

### Sociological data survey

The population in the 3 watersheds is mainly rural (about 60%, [68]), and villages are distributed along major roads (e.g. National Road N13) and waterways (e.g. Nam Khan) (Fig 1). The population density increases with the proximity to the Luang Prabang city: it is of 318 inhabitants per km^2^ in the Houay Xon valley whereas the overall population density in the Nam Khan basin, which includes areas distant from the Luang Prabang city (Fig 1A), is about 13 inhabitants per km^2^. Within the Nam Khan basin, the population density in the Xieng Ngeun district is 21 inhabitants per km^2^.

We conducted a qualitative sociological assessment in order to characterize social representations and hygiene practices dealing with water availability and water quality [69]. The socio-behavioral survey lasted 6 months (June-July 2012 and February-May 2013) but it was not focused on these particular timespans: the interviewees were instead invited to report on habits regarding water sources options when facing usual situations (annual water shortage, floods) and depending on the purpose of water use. The qualitative inventory of perceptions and behaviors was not intended to characterize people who may have been reporting diarrhea cases in the 6 health centers.

The survey consisted of 113 semi-directed interviews with two groups of actors: on the one hand, stakeholders implicated in health management, water supply or sanitation management (31 interviews), and on the other hand, the inhabitants of four villages located in the Luang Prabang area (82 interviews). The stakeholders were selected to get a heterogeneous group of interviewees including non-governmental organization (NGO), public health and environmental administrations, and private companies (S1 Table). The 4 villages (Fig 1B) whose inhabitants were interviewed (S2 Table) were selected in order to be representative of the villages of the Luang Prabang area, according to a criterion of distance and connectivity to the city of Luang Prabang, under the hypothesis that they would not have access to the same sanitation and medical facilities. The 4 villages illustrate contrasted socio-economic situations [70]. Ban Donekang and Ban Napho can be considered as suburban villages, well connected to the city as they are located along the National Roads (N13 and N1, respectively), whereas Ban Long Lao and Ban Xieng Lome are more remote. Villages were also assumed to be subject to contrasting levels of flood damage risk, with usually heavier human, material and economical damage along the Nam Khan (Ban Xieng Lome) and smaller damage along the Houay Xon (Ban Donekang). In each village, interviewees were selected with the help of the head of the village, in order to interview villagers of contrasted socio-economical situations [69]. The “villagers” interview matrix (S3 Table) was composed of 4 sections that gathered information on: (1) the respondents’ occupation and situation, (2) water uses and practices inside and outside the village, (3) flood risk perception and coping strategies, and (4) water related diseases perception and coping strategies. Although the “stakeholders” interview matrix targeted similar information, it was adapted according to the stakeholder activity, putting the emphasis either on public health policies or on water supply and sanitation considerations.

The socio-behavioral assessment relied on the principles of qualitative scientific rigor [71] and did not intend to reproduce a statistical representativeness. Conversely, it intended to represent the diversity of village contexts and villagers’ socio-economical situations that could be encountered in the Luang Prabang area.

### Hydro-meteorological measurements and water sampling

#### Meteorological variables

Climate data were collected from January 1, 2010, to December 31, 2012. Daily rainfall in the Houay Pano catchment was measured by 7 manual rain gauges and an automatic rain gauge (CampbellARG100, 0.2 mm capacity tipping-buckets). To avoid gaps in data records, the average daily rainfall in the Houay Pano catchment was calculated as the mean daily rainfall from the 8 measurements (Fig 1C). Daily air temperature was measured (BaroDIVER probe) at the location of the automatic rain gauge in Houay Pano catchment. Daily rainfall at the Luang Prabang airport was gathered from National Meteorological and Hydrological Center of Lao PDR (Fig 1B).

#### In-stream measurements and samplings

Stream discharge data were collected from January 1, 2010, to December 31, 2012. We calculated the daily discharge of the Nam Khan from water heights recorded twice a day at the NK20 station by the National Meteorological and Hydrological Center of Lao PDR. Stream water level was measured at the S4 outlet with 1 mm vertical precision at a minimum 3-minute time interval by a water level recorder (OTT, Thalimedes) equipped with a data logger within a V-notch weir. A control rating curve (the relationship between water level and discharge) was determined using the velocity area method to calculate discharge at both S4 and NK20.

Samples of stream water (500 mL) were collected manually, approximately 10 cm beneath the water surface, in clean, plastic bottles (i.e. first use after purchase and bottle bag opening) during base flow and high flow approximately twice per month at the S4 and the NK20 outlets from May 2011 to December 2012. In addition, samples were also collected at S4 by an automatic sampler (Automatic Pumping Type Sediment Sampler, ICRISAT), from January 2010 to December 2012. The automatic sampler was triggered by the water level recorder so as to collect water every 2-cm increase during flood rising and every 5-cm decrease during flood recession, approximately 10 cm beneath the water surface.

Temperature of the stream water at S4 and at NK20 was measured biweekly from May 2011 to December 2012 using a Multi Probe System (YSI 556 MPS) at the time of the sampling.

A sub-sample of the collected stream water was used to measure the total suspended sediments (TSS) concentration. TSS was determined for each sample after filtration on 0.2 μm porosity cellulose acetate filters (Sartorius) and evaporation in an oven at 105°C for 48 h. TSS concentration was measured biweekly from May 2011 to December 2012 at the NK20 station and time-continuous from January 2010 to December 2012 at the S4 station.

A second sub-sample was used to determine *E*. *coli* counts. The standardized microplate method (ISO 9308–3) was used for *E*. *coli* counts determinations. Each sample was incubated at four dilution rates (i.e. 1:2, 1:20, 1:200 and 1:2000) in a 96-well microplate (MUG/EC, BIOKAR DIAGNOSTICS) and incubated for 48h at 44°C. Ringers’ Lactate solution was used for the dilutions and one plate was used per sample. The number of positive wells for each microplate was noted and the Most Probable Number (MPN) was determined using the Poisson distribution. This microplate method has previously been used with success at both S4 and NK20 outlets [61,62]. *E*. *coli* numbers were measured biweekly from May 2011 to 2012 at both the NK20 and the S4 stations. However, 3 floods were also sampled at S4 during this period (May 25, 2011; May 15 and June 17, 2012).

#### Groundwater available for streamflow

We used the annual dynamical groundwater volume of storage (*S*_*y*_), which is the water volume available for streamflow, as an indicator of the groundwater level and, hence, of the aquifer water volume available to fill wells. *S*_*y*_ was calculated as:

$$S_{y}=\frac{Q_{0}}{\alpha }\times 86400$$

Where *Q*_*0*_ is the stream base flow discharge (m^3^ s^-1^) at time *t*_*0*_ (January the 1^st^), *α* is the depletion coefficient (d^-1^), characteristic of the groundwater reservoir, estimated by fitting an exponential decay curve [72] to observed stream discharge values during a low flow period without any flood disturbances [73], and 86,400 is a conversion factor from second to day. The values of *S*_*y*_ were then divided by the catchment area to get a specific *S*_*y*_ in mm.

### Data analysis

#### Spatial aggregations

Population statistics in the 5 districts of interest were gathered from the Lao DECIDE Info web portal (www.decide.la accessed in February 2016, based on national population census of 2005). We used the village and district population data to calculate population densities (inhabitants per km^2^) within the polygons of the 5 districts (Luang Prabang, Nan, Phonxai, Phoukhoune, Xieng Ngeun), of the Nam Khan basin, of the Xieng Ngeun district alone, and of the Houay Xon catchment, by summing the settlements populations within each spatial subset polygon.

Population statistics gathered from the Lao DECIDE Info web portal also included the percentage of population aged <5 years old and the sex ratio. We used the population data to calculate diarrhea daily incidences rates per 10,000 inhabitants.

#### Statistical analyses

Some variables (rainfall in Luang Prabang and Houay Pano, air temperature in Houay Pano, discharges and water temperature, both in S4 and NK20, and TSS in S4) were measured daily from January 2010 to December 2012 whereas other variables (water temperature and *E*. *coli* in S4 and NK20, and TSS in NK20) were measured every 2 weeks from May 2011 to December 2012. Here we call “environmental variables” all the variables stemming from hydro-meteorological measurements except *E*. *coli* counts.

Because of the data specificities, statistical analyses were performed in 2 steps. In the first step we investigated the relationships between total incidence and the environmental variables measured daily from 1 January 2010 to 31 January 2012. In a second step we investigated the relationships between *E*. *coli* in S4 and NK20 and the environmental variables measured biweekly at both stations.

In both steps, the relationships were investigated by multivariate generalized linear models using log link and a negative binomial distribution of residuals (in order to account for their over-dispersion). Temporal autocorrelations in the models’ residuals were tested with Durbin-Watson test and a first-order auto-regressive term (ar1) was added to the model in order to correct for such an autocorrelation when necessary. When testing the significances of each variable, potential confounding effects were corrected for by applying a likelihood ratio test (LRT) comparing the full models with and without the variable of interest as described in Faraway (2005) [74].

In the first step of the analysis, the same model was run both on the total 36-month period of the dataset and on the 18-month period from May 2011 to December 2012 that is used in the second step. In the second step, the time-step considered is the one of the water temperature, total suspended sediments and FIB (i.e. 2 weeks). In other words, any entry of the data set containing a missing value was discarded in this second step.

For an exploratory purpose, we calculated Spearman correlation coefficients between temporal variables at both daily time-step (diarrhea incidence, discharge, air temperature, TSS concentration) and biweekly time-step (discharge, water temperature, TSS concentration and *E*. *coli* counts) to identify possible positive and negative associations between variables at both stations.

We also calculated 15 day-moving averages of daily incidences to make it easier to visually identify the diarrhea peaks.

## Results

Dataset is available in supporting information (S4 Table).

### Incidence of diarrheal diseases

A total of 3,367 diarrhea cases were reported from January 2010 to December 2012 across the investigated 5 districts, including 106 in the Xieng Ngeun district, 321 in the Houay Xon valley and 22 in the Laksip village (Table 2). Total annual reported diarrhea cases decreased from 2010 to 2012. The temporal trends were similar for the 3 smaller spatial subsets, although the signal was less clear in the smallest spatial subset (Laksip village) (S1 Fig). The reported daily diarrhea cases in the 4 spatial subsets were positively correlated with each other (Spearman correlation coefficients and p-values are given in S2 Fig). The correlations become stronger when considering 15-day moving averages (S3 Fig).

**Table 2:** Reported diarrheal disease cases (i.e., hospital admissions or incidence) and annual incidence rates (per 10,000 inhabitants) from 2010 to 2012 across Luang Prabang area, Laos, and in the different spatial subsets. The incidence rate over the 2010–2012 period was calculated as the inter-annual average incidence over this period.

	2010–2012	2010	2011	2012
Reported cases	Total 3 years	Total annual	Total annual	Total annual
5 districts	3367	1214	1157	996
Xieng Ngeun district	106	43	46	17
Houay Xon Valley	321	128	118	75
Laksip village	22	12	5	5
**Annual incidence rates (per 10,000 inhabitants)**				
5 districts	60	65	62	53
Xieng Ngeun district	11	13	14	5
Houay Xon Valley	153	183	169	107
Laksip village	149	244	102	102

**Fig 2:**
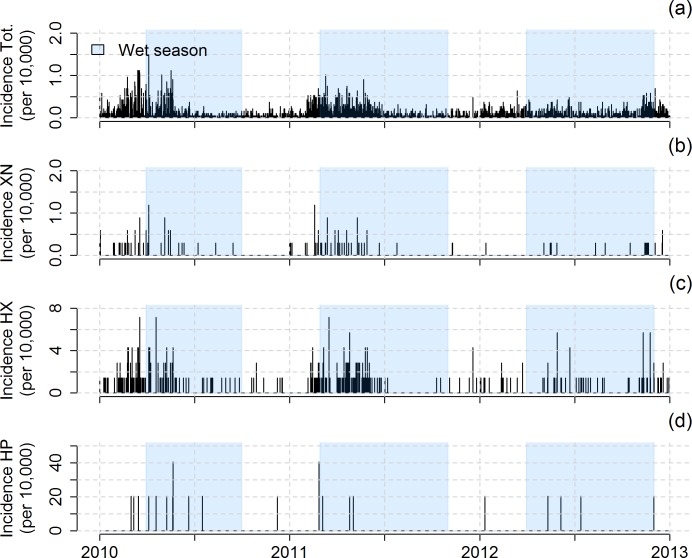
Daily total incidence rates (per 10,000 inhabitants) recorded from 2010 to 2012 across (a) the 5 districts (Total) of interest in this study (Luang Prabang, Nan, Phonxai, Phoukhoune and Xieng Ngeun), (b) the Xieng Ngeun (XN) district including the NK20 sampling station in the Nam Khan river, (c) the 7 villages (Ban Donekang, Ban Khoy, Ban Khuathineung, Ban Laksip, Ban Ma, Ban Phoumork, Ban Sangkhalok) of the Houay Xon (HX) catchment, and (d) Ban Laksip where the S4 sampling station is located in the Houay Pano (HP) river.

Annual incidence rate (i.e. the number of reported cases per 10,000 inhabitants, Table 2) was higher in the nested Houay Xon valley (107–183 cases per 10,000 inhab.) and the Laksip village (102–244 cases per 10,000 inhab.) subsets compared to the Xieng Ngeun district (5–14 cases per 10,000 inhab.). The temporal trends were similar for the 4 spatial subsets (Fig 2).

**Fig 3:**
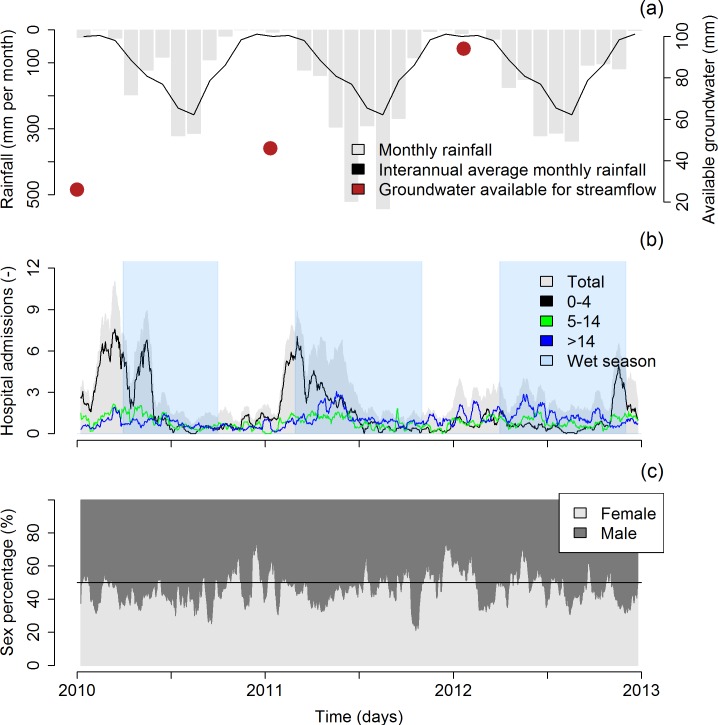
(a) Cumulated rainfall per month (mm) at Luang Prabang, interannual average monthly rainfall (mm) at Luang Prabang and groundwater available for streamflow (mm) in Houay Pano, (b) 15-day moving average of the reported diarrhea cases (i.e., hospital admissions for total, and for the 0–4, 5–14 and >14 age groups), and (c) sex percentage of the diarrhea cases.

Two peaks in hospital admissions occurred in both 2010 and 2011 (Fig 3). In 2010, the first diarrhea epidemic lasted from the end of January to the end of April and the second epidemic lasted from the end of April to the end of May. In 2011, the first diarrhea epidemic lasted from the end of January to the end of March and the second epidemic lasted from the end of March to the beginning of July. In 2012, the temporal pattern was different, the highest peak of hospital admissions occurring from November to December.

In 2010 and 2011, the peaks of diarrhea cases in the >14 age group followed the peaks of the 0–4 age group (Fig 3B). Detailed numbers for each spatial subset are given in supporting information (S5 Table). Regardless of the year, most reported diarrhea cases concerned the 0–4 age group, especially during the incidence peak (Fig 3C). The reported diarrhea cases also mostly concerned male patients (across the 5 districts: +16%, up to 28% for the 0–14 age group). The trends were similar in each spatial subset.

### Hydro-meteorological measurements

**Fig 4:**
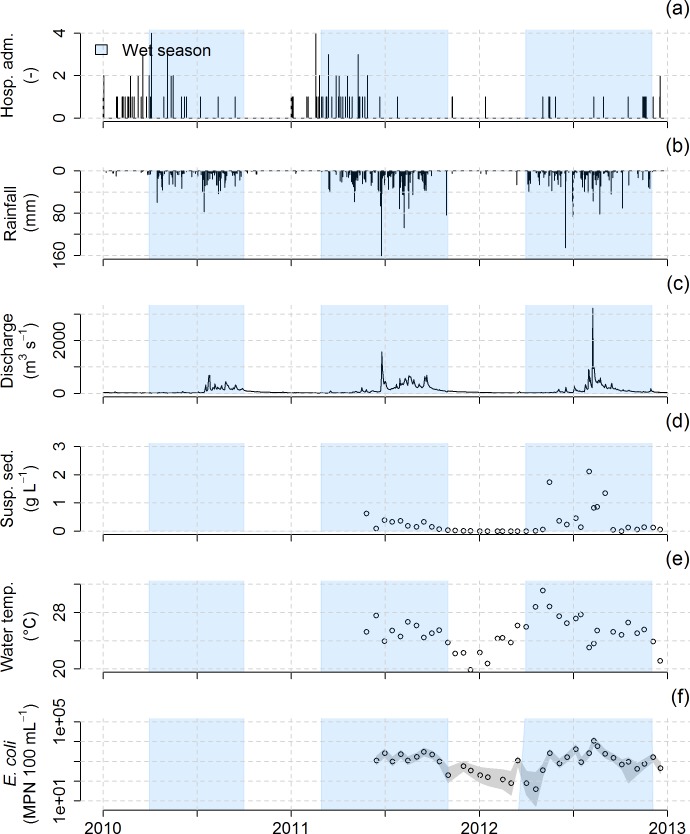
(a) Daily reported diarrheal disease cases (i.e., hospital admissions) recorded in the Xieng Ngeun district, Laos, from 2010 to 2012, (b) Daily rainfall (mm) recorded at the Luang Prabang Airport meteorological station, (c) Daily discharge (m^3^ s^-1^) recorded at the NK20 sampling station, (d) Biweekly monitoring of suspended sediments (g L^-1^) at NK20, (e) Biweekly monitoring of water temperature (°C) at NK20, and (f) Biweekly monitoring of *E*. *coli* counts (MPN 100 mL^-1^) at NK20.

**Fig 5:**
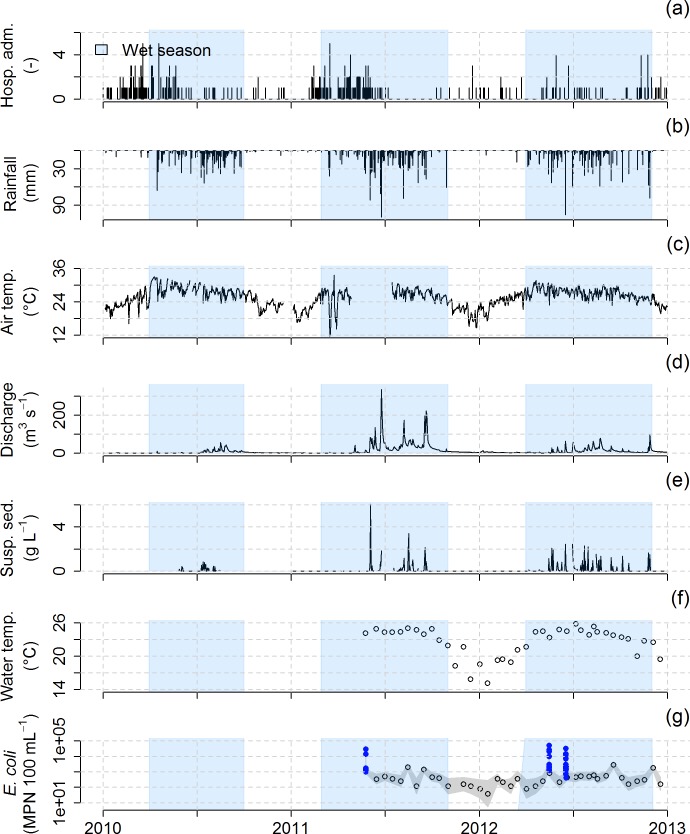
(a) Daily reported diarrheal disease cases (i.e., hospital admissions) recorded across the Houay Xon catchment, Laos, from 2010 to 2012, (b) Daily rainfall (mm) recorded at the Houay Pano meteorological station, (c) Daily air temperature (°C) recorded at the Houay Pano meteorological station, (d) Daily discharge (L s^-1^) recorded at the S4 sampling station, (e) Daily record of suspended sediments (g L^-1^) at S4, (f) Biweekly monitoring of water temperature (°C) at S4, and (g) Biweekly monitoring at S4 of *E*. *coli* counts (MPN 100 mL^-1^, black circles). In addition, *E*. *coli* has been sampled during 3 flood events in 2011 and 2012 (blue points).

In 2010 and 2011, the hydro-meteorological variables were highly seasonal, with different patterns during dry and wet seasons (Fig 4 and Fig 5). In 2010 and 2011, small floods (about 0.005–0.01 m^3^ s^-1^ in S4 and 30–100 m^3^ s^-1^ in NK20) occurred during the dry season, generally from January to March (Fig 3). The temporal pattern of 2012 is less clear, with a wet season associated with more erratic and less intense rainfall. The estimated dynamical volume of storage (water in the saturated zone) at S4 on January 1^st^ was of 26 mm in 2010, of 46 mm in 2011, and of 94 mm in 2012 (Fig 3A).

Each year, the air temperature dropped to a minimum (~15°C) in December-January whereas maximum temperatures of 25–30°C were recorded from May to September. Water temperature followed the same temporal trend with minimum temperatures of 20°C and 16°C for NK20 and S4, respectively, and maximum temperatures of 28°C and 24°C for NK20 and S4, respectively. The seasonal pattern of *E*. *coli* counts was less clear, being high all year long, although slightly higher during the wet seasons compared to the dry seasons. Overall, the biweekly measurements showed a baseline of 1,516±2,031 MPN 100 mL^-1^ at NK20 and 478±583 MPN 100 mL^-1^ at S4, whereas *E*. *coli* counts during floods sampled at S4 rose to 7,469±11,266 MPN 100 mL^-1^.

Rainfall in Luang Prabang and Houay Pano, discharge in S4 and NK20, and suspended sediments in S4 were positively correlated with each other (S2 Fig). Air temperature was also positively correlated with rainfall in Luang Prabang and Houay Pano. At both S4 and NK20, discharge was positively correlated with suspended sediment concentrations and *E*. *coli* counts (S4 Fig). Water temperature was positively correlated to discharge and other variables at S4 but no correlation was found between the water temperature and the other hydro-meteorological variables in NK20.

### Relationship between diarrhea incidence and hydro-meteorological variables

Overall, the reported cases of diarrhea in the 4 spatial subsets were negatively correlated to discharge at both NK20 and S4 (S4 Fig and S3 Fig).

#### Effect of environmental variables on diarrhea incidence

The negative binomial multivariate model showed that the total incidence significantly increased with air temperature in Houay Pano and decreased with discharge in NK20—and marginally with discharge in S4 (Table 3). When running the same model on the May 2011-December 2012 period, none of the variables had any significant effect (S6 Table).

**Table 3:** Effect of environmental variables on total incidence from 1 January 2010 to 31 December 2012. The last three columns present the results of the LRT and show the significances of each variable, correcting for the potential confounding effects of the other variables. ar1 is the 1-step lagged incidence variable.

	Estimate	Std. Error	z value	Pr(>|z|)	Df	Deviance	Pr(>Chi)
(Intercept)	0.3434	0.2285	1.5030	0.1328	-	-	-
ar1	0.1060	0.0079	13.3393	<0.0001	-	-	-
Rainfall_LP	-0.0049	0.0046	-1.0581	0.2900	1	1.0855	0.2975
Rainfall_HP	0.0007	0.0050	0.1313	0.8956	1	0.0163	0.8984
Air_Temp_HP	0.0186	0.0091	2.0457	0.0408	1	4.2759	0.0387
Discharge_S4	-0.0038	0.0028	-1.3203	0.1867	1	2.1937	0.1386
Discharge_NK20	-0.0010	0.0004	-2.7059	0.0068	1	10.5491	0.0012

#### Effect of environmental variables on *E*. *coli* counts

*E*. *coli* counts increased with water temperature in S4 (Table 4) but no environmental variable had any significant effect on *E*. *coli* counts in NK20 (Table 5).

**Table 4:** Effect of environmental variables on *E*. *coli* counts in S4 from May 2011 to December 2012. The last 3 columns present the results of the LRT and show the significances of each variable, correcting for the potential confounding effects of the other variables. No auto-regressive coefficient was included in the model since no significant temporal auto-correlation was detected by the Durbin-Watson test.

	Estimate	Std. Error	z value	Pr(>|z|)	Df	Deviance	Pr(>Chi)
(Intercept)	1.2395	1.3481	0.9195	0.3579	-	-	-
Rainfall_LP	0.1065	0.0754	1.4129	0.1577	1	1.4118	0.2348
Rainfall_HP	-0.1528	0.0798	-1.9159	0.0554	1	2.9548	0.0856
Air_Temp_HP	-0.0451	0.0908	-0.4969	0.6192	1	0.2783	0.5978
Discharge_S4	0.0209	0.0205	1.0203	0.3076	1	0.6371	0.4248
TSS_S4	-1.0955	2.1494	-0.5097	0.6103	1	0.2078	0.6485
Water_Temp_S4	0.2522	0.1275	1.9773	0.0480	1	4.8901	0.0270

**Table 5:** Effect of environmental variables on *E*. *coli* counts in NK20 from May 2011 to December 2012. The last 3 columns present the results of the LRT and show the significances of each variable, correcting for the potential confounding effects of the other variables. ar1 is the 1-step lagged FIB variable.

	Estimate	Std. Error	z value	Pr(>|z|)	Df	Deviance	Pr(>Chi)
(Intercept)	4.0848	2.2692	1.8001	0.0718	-	-	-
ar1	0.0001	0.0001	0.9215	0.3568	-	-	-
Rainfall_LP	0.0371	0.0599	0.6191	0.5359	1	0.4144	0.5198
Rainfall_HP	-0.0207	0.0752	-0.2758	0.7827	1	0.0690	0.7928
Air_Temp_HP	0.1480	0.1354	1.0928	0.2745	1	0.9688	0.3250
Discharge_NK20	0.0012	0.0018	0.6650	0.5061	1	0.5477	0.4593
Water_Temp_NK20	-0.0704	0.1916	-0.3673	0.7134	1	0.0999	0.7520
TSS_NK20	1.1957	0.6777	1.7642	0.0777	1	2.2411	0.1344

### Villagers’ ordinary practices and social perceptions of water contamination hazards

The socio-behavioral survey showed that decision-makers and politics consider rural areas at risk for diarrheal diseases. The rivers are mostly used as sewers because efficient sanitation and garbage treatment facilities are lacking. From a medical perspective, the “Sam Saat” national awareness health campaigns to avoid diarrhea are organized at the beginning of the hot season. “Sam Saat” campaigns, the 3 pillars of which are “eat clean, drink clean, stay clean”, mostly focus on individual hygiene practices within the households and on diseases related to the ingestion of water (drinking water), thus omitting diseases specifically related to skin contact to water (e.g. dish-washing, laundry, body cleaning, bathing in rivers) and to the ingestion of pathogens from oral-skin contact (e.g. children sucking their hands, breast feeding).

Villagers take water from various sources depending on water use (Table 6). They have more water source options within the village than in the fields. Hence, they have more options to cope with potential water contamination within the village than outside. Villagers actually rank the various contamination paths related to waterborne diseases, and for a given water use, they take what they consider to be the safest water source(s) as primary water source(s) (Table 6, rank 1). Drinking water is seen as the water use requiring the most control to avoid diseases. For drinking purposes, villagers mostly rely on bottled water and improved water sources (municipal water fountains, protected wells and sources). However, more remote villages do not purchase bottled water, and villagers working in the field do not always bring enough bottled water and need to rely on non-improved water sources (river water, non-protected wells, water tanks on carts). Villagers also mostly use improved water sources for household and body cleaning while they mostly rely on non-improved water sources for other uses (including recreational use). Surface water, or river water, is used for recreational use (e.g. children’s games and bathing): even if villagers consider that river water is of lower quality, they do not consider skin contact with river water as a health threat. In addition, younger children may be totally unaware of any health risk and drink river water when playing in the river.

**Table 6:** Water sources options depending on water uses in the Luang Prabang rural area. Water sources options are ranked from 0 to 3, 0 meaning that the water source is not relevant for the considered use, 1 being the preferred water source for the water use considered, provided it is financially or technically available, 2 being the alternative water source when option 1 is not available (e.g. a temporary shortage of bottled water) and 3 being the second alternative water sources, when both options 1 and 2 are not available (e.g. when the municipal water fountain runs out of water during the hot season, or when it breaks down because of limestone deposits in pipes). Two ranks given for one water source means that what is considered as an alternative water source for some villagers can be considered as the main water source for others.

		Water source within the village			Water source outside the village		
Water uses		Bottle	Improved water source*	Non-improved water source**	Bottle	Improved water source*	Non-improved water source**
**Drinking**		1	2	3	1	0	1, 2
**Domestic uses**	Dish washing	0	1	1, 2	0	0	1
	Laundry	0	1	1, 2	0	0	1
	Housework	0	1	1, 2	0	0	1
	Body cleaning	0	1	1, 2	0	0	1
	Toilet flushing	0	1	1, 2	0	0	1
**Recreation, fishing**		0	0	1	0	0	1
**Cattle**		0	0	1	0	0	1
**Irrigation, agriculture**		0	0	1	0	0	1
**Fish farming**		0	0	1	0	0	1

* Protected wells and sources, municipal water fountains

** River, temporary non-protected wells

Water sources can also be considered as the main water source by some villagers whereas they can be considered as an alternative water source by some other villagers (e.g. non-improved water source for domestic uses in villages, Table 6). These situations are rather rare and are mostly due to either insufficient household financial capacities, or when the house is considered to be too far from the improved water source, so that the villagers chose the closest water source even if it perceived as less safe.

Each year, villagers swap water sources depending on availability, location (i.e. when they stay in or nearby the village or when they carry out field work), and water quality perception. When water conveyance is defective or when the source is scarce (e.g. dry municipal water fountain during the hot season, broken pipes due to excessive limestone deposits), villagers need to switch their water supply to a secondary water source that they consider less safe, such as non-improved water sources. When all other sources are dry, the river still flows and appears as the best option to meet most of the water needs.

Interestingly, villagers consider their surroundings inside and outside the village as a space they know and control, in which they evaluate water quality based on visual (e.g. water color) and olfactory (e.g. putrefaction or chlorine) indicators. However, they do not consider that the sewage they release daily into the environment contributes to the fecal contamination of the river, nor that it decreases surface water quality and increases health risks. In addition, villagers do not consider themselves more at risk when working on the field. This contrasts with stakeholders’ perceptions, which precisely consider the field as risky area where people are more likely to be exposed to water contamination, because of the lack of improved water sources (including cooking facilities to boil water) and of sanitation facilities.

## Discussion

The temporal pattern of reported diarrhea cases in the smaller spatial subsets was similar to the overall pattern (5 districts), even though they do not belong to the same drainage area. This allowed us to consider only total incidence (5 districts) in the multivariate analysis that showed that total incidence increased with air temperature in Houay Pano but decreased when discharge in NK20 (and to a lesser extent in S4) increased. However, when running the same model on the May 2011-December 2012 period, none of the variables had any significant effect. This is not surprising given that the strong seasonality observed in the diarrhea epidemiology in 2010 and 2011 vanished dramatically in 2012. Multivariate analysis also showed that *E*. *coli* counts increased with water temperature in S4 whereas nothing seemed to affect *E*. *coli* counts in NK20. The fact that a significant result is observed in S4 and not in NK20 could be due to the size of the respective catchments: there may be less heterogeneity in small catchments than in large ones and thus the relationships between variables may be stronger in smaller catchments.

Unfortunately, the seasonality of the diarrhea epidemiology vanished dramatically since we started measuring *E*. *coli* in the Luang Prabang area. This renders difficult relating environmental, FIB and epidemiological variables in one single multivariate model. Consequently, we had to perform two successive analyses on two different time periods and involving different variables. The first focused on the link between environmental variables and incidence and showed a positive effect of air temperature and a negative effect of discharge. The second analysis focused on the link between environmental variables and FIB and showed a positive effect of water temperature. Of note, water temperature could not be included in the first analysis but the air temperature variable could be considered as a good proxy of water temperature in this first analysis (the two being strongly correlated, Spearman correlation coefficient is 0.84, p-value<0.001). Thus, the positive effect of water temperature on *E*. *coli* and of air temperature on diarrhea incidence could be interpreted as an increase of *E*. *coli* with water temperature and an increase of diarrhea infection with increased *E*. *coli*. The fact that discharge has a negative effect on diarrhea incidence and no effect on FIB suggests that the effect of discharge is more related to human behavior than to the biology of the potential etiologic agent. Inconclusive associations between indicator bacteria in drinking water and disease risk have already been reported [25,44,45]. Associations between diarrheal infections and meteorological parameters such as rainfall and temperature are not uniform across climates zones and time periods, highlighting a complex relationship between weather, water quality, human behavior and waterborne diseases [25,31].

Several authors have attempted to identify a putative linkage between the incidence of diarrhea and environmental and anthropogenic drivers. We showed that the reported hospital visits due to diarrhea in the Luang Prabang area, Laos, were positively correlated to air temperature and negatively correlated to river discharge (Table 3) and that they followed a bimodal trend, with the first epidemic (highest peak) starting at the end of the dry and the hot season and ending at the beginning of the wet season, and the second epidemic occurring during the wet season.

The first diarrhea epidemic (January-April in 2010, January-March in 2011 and to a lesser extent January in 2012) is consistent with the results of previous studies in Laos [13] and in other tropical areas such as the Northeast of Thailand [28,48], the Pacific Islands [31], Bangladesh [34], Nepal [26] and Sub-Saharan Africa [29,30]. Some of these studies related the triggering of the diarrhea epidemic to water shortages and the use of alternative, informal water sources [26,29–31]. In addition, several authors found bimodal peaks of diarrhea incidence, associated with peaks of etiologic agents at different times of the year [25,29,34,48,75]. For instance, Phetsouvanh et al. (1999) found that annual diarrhea incidence among young children in the Vientiane area, Laos, was bimodal with peaks in February and June that were associated to rotavirus and bacteria, respectively [13]. The peak of childhood diarrhea in January-March (Fig 3B) also supports this pattern of etiologic agent dynamics, as rotavirus is generally more common in young children than adults [8,76].

In the Luang Prabang area, most of the villages use municipal water fountains for their domestic water supply. These water fountains are connected to wells. However, in the absence of rainfall, e.g. at the end of the dry season, water levels in the wells may drop below the pumping threshold, thus inducing a water shortage [77]. In this situation, the villagers tend to switch their water supply to surface water. Moreover, during the dry season surface water has lower turbidities, giving the impression that the water is “clean”. As a result, the waterborne disease risk perception by the population is low. Also, villagers do not consider that they contribute to the fecal contamination of the river. This may be due to the absence of collective sewage management and to the lack of perceptions of individual contributions to river pollution. However, our results presented here and in other work from this area [61–63] show that *E*. *coli* counts are high throughout the year, and although slightly lower during the dry season, they always exceed the WHO recommendation of 0 MPN 100 mL^-1^ in drinking water [40]. In the Luang Prabang area, people mostly use bottled water as drinking water, but they use untreated, contaminated surface water for other domestic activities such as washing cooking utensils, vegetables and fruit, laundry and bathing. Higher *E*. *coli* counts during floods at the end of the dry and hot season (about 10,000 MPN 100 mL^-1^ at S4, Fig 5G) may be explained by the wash-off of animal and human dejections at soil surface and by the overflowing of latrines [61].

We observed that the first diarrhea epidemics ended when the wet season started. Some authors suggested that the end of the epidemic could be driven by host immunity [51] and/or by the exhaustion of bacterial stores due to the continuous rainfall flushing [31,78]. The latter was not clear in our data as the baseline of *E*. *coli* counts was high throughout the year. Hence, we propose that the end of the first epidemics in the Luang Prabang area may be attributed to both host immunity (susceptible depletion) and to an increase in water level in the municipal wells, and hence, to a switch back to well pumping as a primary water source. The estimated dynamical volume of groundwater available for streamflow each year on January 1, used here as a proxy of groundwater stock, supports the hypothesis that diarrhea epidemics are driven by water shortage. Interestingly, the dynamical groundwater volume on January 1^st^ increased from 2010 to 2012, suggesting that groundwater availability increased over the study period. A higher water table increases water availability in wells and municipal water fountains and contributes to higher discharges, thus diluting the constant input of fecal material. This may partly explain why diarrhea incidence decreased from 2010 to 2012. An additional decreasing factor could be the success of annual national awareness health campaigns [69,79].

The second epidemic of diarrhea (April-May in 2010 and March-July in 2011) starting and ending during the wet season is consistent with the positive associations found between diarrhea incidence and rainfall [16,20,24,25] and between diarrhea incidence and water level increase [21,33,34]. It is also consistent with the peak of the bacterial causal agent reported by Phetsouvanh et al. [13]. Here the end of the epidemic may be driven either by host immunity [51] and/or by the exhaustion of bacterial stores due to the continuous rainfall flushing [31,78].

The over-representation of young children among patients is already well documented [28,52]. This is due to their higher vulnerability to the effects of climate on diarrheal illness, as they may be highly exposed (via close contact with the environment) and highly sensitive (because of their relatively poorly developed immunity and their weaker body water retention capacity) [80]. A second explanation may be the propensity of young children to bathe and play in ephemeral streams during small flood events occurring during the dry and hot season, leading to relatively higher *E*. *coli* counts (Fig 5G). A third explanation can be oral contamination associated with breast feeding if the mother washed herself with contaminated water. The over-representation of male patients, mostly young male children, can be attributed to the fact that they are more frequently playing outside the households and have higher risk behavior compared to young female children [81].

The similar temporal patterns observed for the 5 districts and for the Xieng Ngeun district suggest that the dynamics of the diarrheal disease occurrence is independent of population density. Moreover, the similar temporal patterns in space also suggest that small spatial subsets are representative subsets of the overall incidence over larger areas, thus limiting the extent of future epidemiological surveys. This also highlights that the results of the statistical analyses made on the total incidence (5 districts) are valid whatever the considered drainage area, and that the comparison of diarrhea incidences in Xieng Ngeun and Houay Xon/Houay Pano together with hydro-meteorological variables in NK20 and S4, respectively, and with the socio-behavioral dataset (Table 1), is relevant. However, although the dynamic was similar, the magnitude of incidence rate variations was different depending on the spatial subset. Incidence rate was actually lower in the less densely populated district of Xieng Ngeun and higher in the more densely populated area closer to the Luang Prabang city (e.g. Houay Xon catchment). The overall incidence rate in the Luang Prabang district is 401 cases per 10,000 inhabitants, i.e. 13 times higher than the incidence in the Xieng Ngeun district. Differences in diarrhea incidence between urban and rural areas have been highlighted by several studies in the tropics [20,25,75]. These studies concluded that the association between climate factors and diarrhea was stronger in rural than in urban areas and that urban areas were better able to dampen the seasonal variability of diarrheal diseases as driven by climate. Also, urban areas were often associated to higher GDPs and higher educational attainments, and they had greater investments in sanitation and thus were better able to dispose fecal material [28,34,75,82]. Hence, none of these studies have shown higher incidence rates of diarrhea in more densely populated areas as we found in the Luang Prabang area. Manifold explanations may be suggested here: cases may be more often reported in urban areas rather than in rural areas because (1) the access to the health facilities is easier, (2) the population living close to urban areas may be wealthier and can afford medication, (3) the population living close to urban areas are more aware of the benefits from modern medicine [79,83] and (4) the probability of contagion is higher in more densely populated areas.

These latter explanations also raise the possible issue of the quality of the hospital admission dataset. Most of the diarrhea cases that were reported to the 6 health centers in the Luang Prabang area may have been moderate or severe hospital-admitted cases, as people with mild symptoms may not seek medical care. Therefore, we may have underestimated the cases of minor diarrhea. In Laos people can easily buy medicine in any private pharmacy or grocery store to treat themselves or their children without hospital admission; however, the focus on data from hospital admissions provides a more accurate measure of more serious illness [16,21]. Also, diarrhea cases during the wet season may be under-reported since remote villagers may not be able to access health centers because roads are often blocked due to flooding and landslide.

Additional limitations of our study are related to the daily counts of diarrhea cases that were mostly clinically defined and not biologically assessed. Hence, counts combined all causes, so we were not able to analyze cause-specific diarrhea, i.e. disease etiology. This may reduce the magnitude of the hydro-meteorological/diarrhea relationship because microbiological agents interact with weather factors [21]. Compilation of cases with multiple causal agents may also distort the assessment of peak timing [84,85], however a distortion of the assessment of the peak timing does not really affect our conclusion here that the 1^st^ peak of diarrhea is triggered in the dry season and that the 2^nd^ peak is triggered in the wet season. Finally, this study was limited in analyzing the sensitivity of some potential confounding factors such as household economic level, vaccination coverage, immune system and dietary habits due to a lack of data on these factors [52].

Conversely, one of the strengths of our study relates to the catchment approach that integrates the heterogeneity of rainfall, soil and land use (the latter including both land cover and management practices, e.g. land used for open defecation) upstream of the monitoring stations. In Northern Laos, villages and communication pathways such as roads (e.g. N13) and waterways (e.g. Nam Khan) are located in the valleys. Hence, the catchment approach also appears to be a relevant spatial unit since people are living close to the possible water quality monitoring points (e.g. S4 and NK20). In addition, previous studies during both low and high flow in the Houay Pano, Houay Xon and Nam Khan catchments showed that *E*. *coli* counts were high all along the rivers, with a slight increase from upstream to downstream [61,63]. Hence, we can consider that water quality at both S4 and NK20 was representative of water quality all along the river, regardless of discharge. In the context of climate change, annual rainfall in South-East Asia is expected to increase [86]. However, combined to the expansion of higher evaporative demand perennial crops, groundwater recharge may decrease [87] and be less available for human needs. Concomitantly, more intense rainfall and land use changes towards annual crops or trees without understorey may increase runoff and decrease water travel times downstream, thereby reducing the filtration process of pathogens before entering the stream.

### Conclusion

This study is the first to deal with diarrhea occurrence in the northern montane area of the Lao PDR and it is an addition to the rapidly growing body of literature attempting to associate reported diarrhea cases to socio-demographic factors and to a range of hydro-meteorological variables on the spatial scale of a whole river basin.

We found that the diarrhea epidemics in the Luang Prabang area started during the hot and dry season and ended during the wet season. The fact that *E*. *coli* counts are high all year long at the two monitored stations indicates that fecal contamination is ubiquitous and constant. This suggests that *E*. *coli* may not be a relevant indicator of the overall fecal risk in this area. Our results suggest that water shortage triggers the 1^st^ diarrhea peak during the dry season, and that rainfall and aquifer refill ends the epidemic during the wet season. These results also suggest that anthropogenic drivers, such as risk awareness and hygiene practices, are at least as important as environmental drivers in determining the seasonal pattern diarrhea epidemic [70].

Similar environmental forcing showed similar dynamics of the occurrence of diarrhea independently of the spatial unit considered (e.g. several villages or a whole district). Smaller spatial subsets were thus representative of the larger ones. For diarrheal diseases risk monitoring, the catchment monitoring approach is relevant because it allows integrating both rainfall and land use, and consequent surface water hydrology, over a large spatial area, since rainfall and land use can be very heterogeneous in large catchments such as the Nam Khan basin. However, the frequency of the measurement of a diarrhea incidence proxy should be adapted to the catchment size. For instance, bi-weekly monitoring of small basins such as the nested Houay Xon and Houay Pano catchments may not be frequent enough to capture floods which last less than a day, whereas it might be adapted to the hydrology of the Nam Khan or to the Mekong. The negative correlations between discharge and reported diarrhea cases suggest that discharge, or groundwater level, can be considered as relevant proxies for diarrhea epidemics and that changes in discharge could be used as an essential monitored variable in an early warning system.

Mitigating both environmental and anthropogenic drivers of diarrhea epidemics appears especially critical in a global change context. From a land management point of view, riparian vegetation should be favored for its filtering service. Finally, instead of focusing only on individual hygiene practices, national health policies should also make the population aware that individuals belong to a community, and more broadly to an ecosystem, implying interactions between the population and its environment.

## Supporting Information

S1 Fig Daily total reported diarrheal diseases cases (i.e., hospital admissions or “Hosp. adm.”) recorded from 2010 to 2012 across (a) the 5 districts (Total) of interest in this study (Luang Prabang, Nan, Phonxai, Phoukhoune and Xieng Ngeun), (b) the Xieng Ngeun (XN) district including the NK20 sampling station in the Nam Khan river, (c) the 7 villages (Ban Donekang, Ban Khoy, Ban Khuathineung, Ban Laksip, Ban Ma, Ban Phoumork, Ban Sangkhalok) of the Houay Xon (HX) catchment, and (d) Ban Laksip where the S4 sampling station is located in the Houay Pano (HP) river.
(PDF)

S2 Fig Spearman coefficient of correlation between reported diarrheal diseases cases (i.e., hospital admissions or incidence) at 4 spatial subsets (See supplementary information S1 Fig for full details), daily rainfall (mm) records at two locations (Luang Prabang Airport and Houay Pano catchment) and daily air temperature (°C) record across the Houay Pano catchment, daily discharge records at two gauging stations (NK20 on the Nam Khan in m^3^ s^-1^ and S4 on the Houay Pano in L s^-1^) and daily total suspended sediments (g. L^-1^) records at the S4 sampling station, from 2010 to 2012. (PDF)

S3 Fig Spearman coefficient of correlation between 15-days moving average of reported diarrheal diseases cases (i.e., hospital admissions or incidence) at 4 spatial subsets (See S1 Fig for full details), daily rainfall (mm) records at two locations (Luang Prabang Airport and Houay Pano catchment) and daily air temperature (°C) record across the Houay Pano catchment, daily discharge records at two gauging stations (NK20 on the Nam Khan in m^3^ s^-1^ and S4 on the Houay Pano in L s^-1^) and daily total suspended sediments (g. L^-1^) records at the S4 sampling station, from 2010 to 2012. (PDF)

S4 Fig Spearman correlation coefficients between water temperature (°C), discharge (m^3^ s^-1^), total suspended sediments (g L^-1^) and *E*. *coli* counts (MPN mL^-1^) measured from 2011 to 2012 at (a) the NK20 gauging station in the Nam Khan basin, and (b) at the S4 gauging station in the Houay Pano catchment.
(PDF)

S1 Table Detail of the stakeholders interviewed. (PDF)

S2 Table Detail of the villagers interviewed. (PDF)

S3 Table Villagers interview matrix. (PDF)

S4 Table Dataset. (XLSX)

S5 Table Reported cases (i.e., hospital admissions) of diarrheal diseases from 2010 to 2012 across Luang Prabang area, Laos, by (1) the age of the patients, (2) their sex and (3) the spatial subset. (PDF)

S6 Table Effect of environmental variables on total incidence from May 2011 to December 2012. The last 3 columns present the results of the LRT and show the significances of each variable, correcting for the potential confounding effects of the other variables. ar1 is the 1-step lagged incidence variable.
(PDF)
