# Supplementary material for: Hydrological Regime and Water Shortage as Drivers of the Seasonal Incidence of Diarrheal Diseases in a Tropical Montane Environment
Source: PLoS Negl Trop Dis. 2016 Dec 9;10(12):e0005195. doi: 10.1371/journal.pntd.0005195 (PMC5147807; doi:10.1371/journal.pntd.0005195)
Supplement: S1 Fig — Daily total reported diarrheal diseases cases (i.e., hospital admissions or “Hosp. adm.”) recorded from 2010 to 2012 across (a) the 5 districts (Total) of interest in this study (Luang Prabang, Nan, Phonxai, Phoukhoune and Xieng Ngeun), (b) the Xieng Ngeun (XN) district including the NK20 sampling station in the Nam Khan river, (c) the 7 villages (Ban Donekang, Ban Khoy, Ban Khuathineung, Ban Laksip, Ban Ma, Ban Phoumork, Ban Sangkhalok) of the Houay Xon (HX) catchment, and (d) Ban Laksip where the S4 sampling station is located in the Houay Pano (HP) river. (PDF) [file pntd.0005195.s001.pdf]

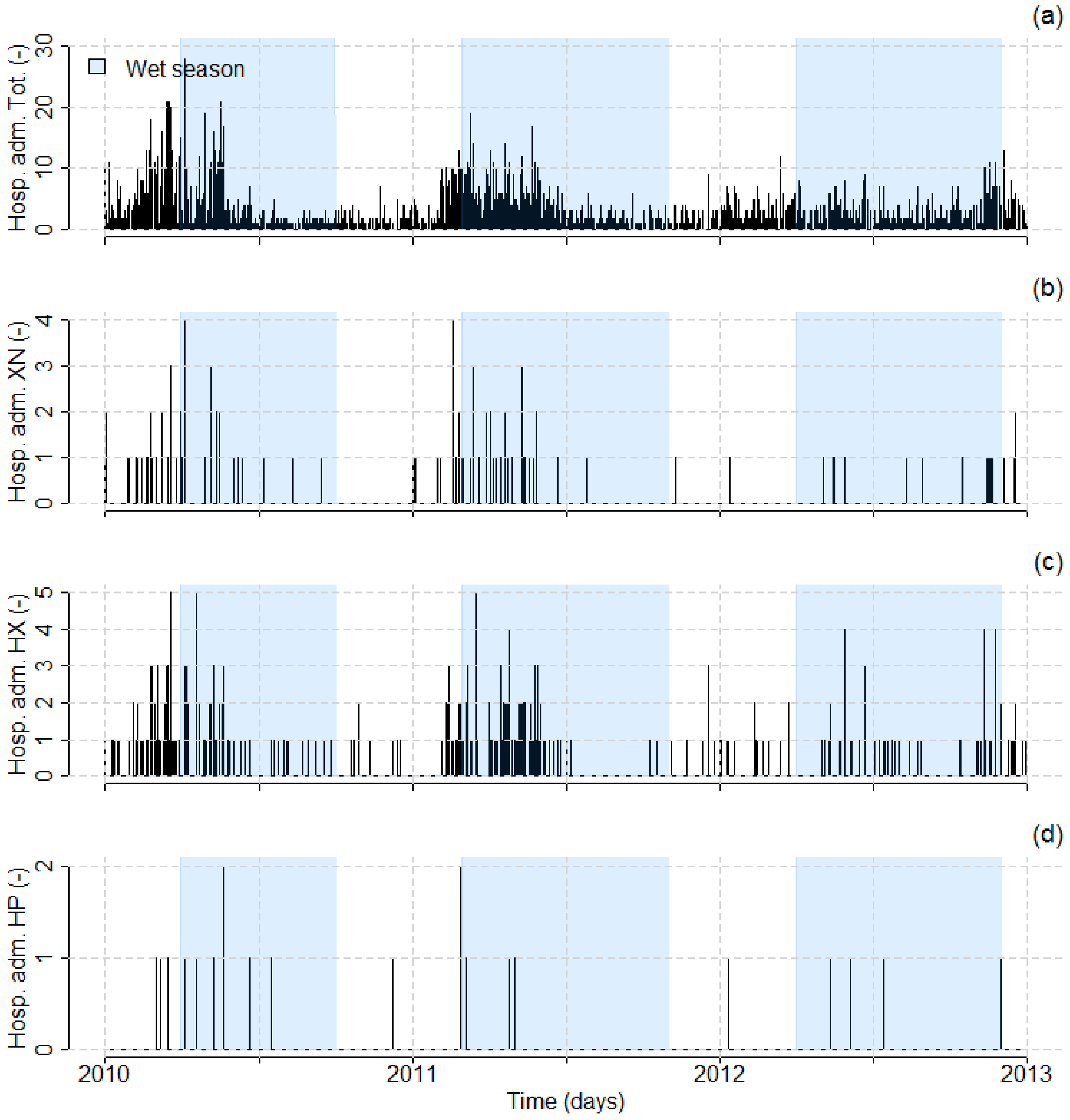

S1 Fig. Daily total reported diarrheal diseases cases (i.e., hospital admissions or "Hosp. adm.") recorded from 2010 to 2012 across (a) the 5 districts (Total) of interest in this study (Luang Prabang, Nan, Phonxai, Phoukhoune and Xieng Ngeun), (b) the Xieng Ngeun (XN) district including the NK20 sampling station in the Nam Khan river, (c) the 7 villages (Ban Donekang, Ban Khoy, Ban Khuathineung, Ban Laksip, Ban Ma, Ban Phoumork, Ban Sangkhalok) of the Houay Xon (HX) catchment, and (d) Ban Laksip where the S4 sampling station is located in the Houay Pano (HP) river.
