# Supplementary material for: Hydrological Regime and Water Shortage as Drivers of the Seasonal Incidence of Diarrheal Diseases in a Tropical Montane Environment
Source: PLoS Negl Trop Dis. 2016 Dec 9;10(12):e0005195. doi: 10.1371/journal.pntd.0005195 (PMC5147807; doi:10.1371/journal.pntd.0005195)
Supplement: S3 Fig — (PDF) [file pntd.0005195.s003.pdf]

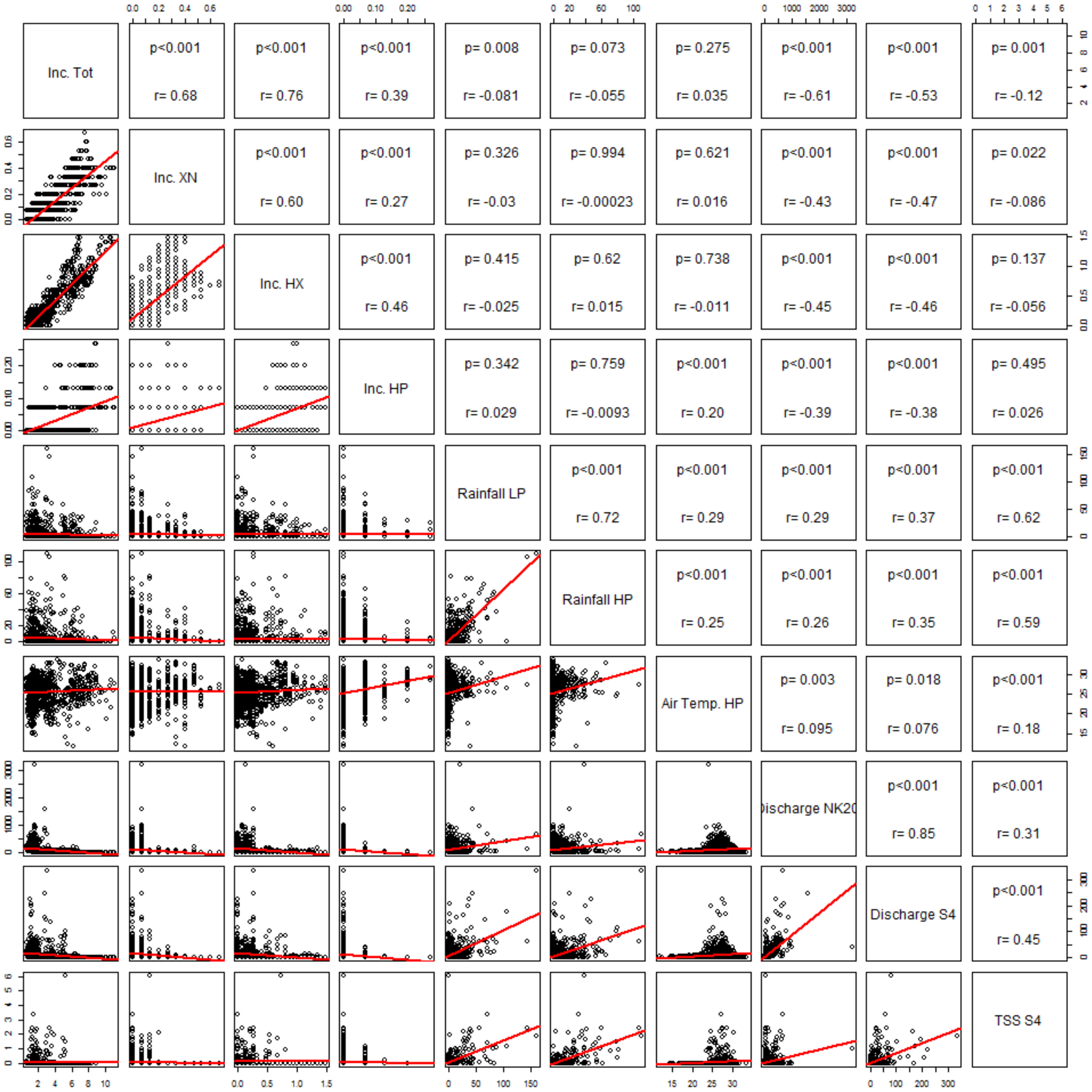

S3 Fig. Spearman coefficient of correlation between 15-days moving average of reported diarrheal diseases cases (i.e., hospital admissions or incidence) at 4 spatial subsets (See Fig. SI1 for full details), daily rainfall (mm) records at two locations (Luang Prabang Airport and Houay Pano catchment) and daily air temperature (°C) record across the Houay Pano catchment, daily discharge records at two gauging stations (NK20 on the Nam Khan in $m^3 s^{-1}$ and S4 on the Houay Pano in $L s^{-1}$) and daily total suspended sediments ($g. L^{-1}$) records at the S4 sampling station, from 2010 to 2012.
