# Supplementary material for: Hydrological Regime and Water Shortage as Drivers of the Seasonal Incidence of Diarrheal Diseases in a Tropical Montane Environment
Source: PLoS Negl Trop Dis. 2016 Dec 9;10(12):e0005195. doi: 10.1371/journal.pntd.0005195 (PMC5147807; doi:10.1371/journal.pntd.0005195)
Supplement: S4 Fig — Spearman correlation coefficients between water temperature (°C), discharge (m3 s-1), total suspended sediments (g L-1) and E. coli counts (MPN mL-1) measured from 2011 to 2012 at (a) the NK20 gauging station in the Nam Khan basin, and (b) at the S4 gauging station in the Houay Pano catchment. (PDF) [file pntd.0005195.s004.pdf]

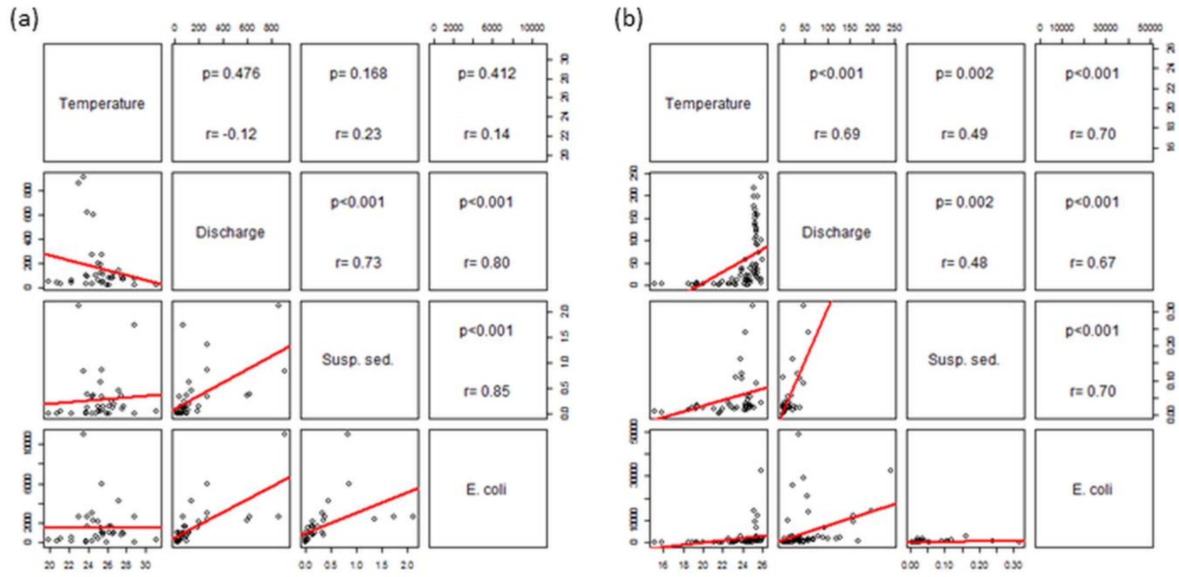


S4 Fig. Spearman correlation coefficients between water temperature (°C), discharge ($m^3$ $s^{-1}$), total suspended sediments (g $L^{-1}$) and *E. coli* counts (MPN $mL^{-1}$) measured from 2011 to 2012 at (a) the NK20 gauging station in the Nam Khan basin, and (b) at the S4 gauging station in the Houay Pano catchment.
