# Supplementary material for: Hydrological Regime and Water Shortage as Drivers of the Seasonal Incidence of Diarrheal Diseases in a Tropical Montane Environment
Source: PLoS Negl Trop Dis. 2016 Dec 9;10(12):e0005195. doi: 10.1371/journal.pntd.0005195 (PMC5147807; doi:10.1371/journal.pntd.0005195)
Supplement: S1 Table — (PDF) [file pntd.0005195.s005.pdf]

S1 Table. Detail of the stakeholders interviewed.

**Group of stakeholders**	**Stakeholders**	**Number of interviewees**	**Additionnal information**
Health administrations	Provincial hospital	5	Various actors : Administrators, doctors, nurses, pharmacy owners
	District hospital	2	
	Dispensary	4	
	Pharmacies	2	
	Public Health Administration	3	
Water management and engineering	NGOs and international investors	8	GRET*, French Red Cross*, Lao Red Cross, World Vision, Confluences, World Fund*, GTZ, LIRE*, AFD*
	Companies	4	Luang Prabang sewage draining company, Water Technical Assistance* (water conveyance and wastewater engineering), Terra Clear* (water treatment solutions), DSK group (bottling company)
	Public Administrations	2	Luang Prabang Administration (on waste management), Nam Papa (urban water conveyance), PAFO (agriculture)
** These stakeholders are based in Vientiane Capital and do not necessarily work in Luang prabang District. However, they provided useful insights on water and/or on health and sanitation stakes in rural areas.*			
