# Supplementary material for: Hydrological Regime and Water Shortage as Drivers of the Seasonal Incidence of Diarrheal Diseases in a Tropical Montane Environment
Source: PLoS Negl Trop Dis. 2016 Dec 9;10(12):e0005195. doi: 10.1371/journal.pntd.0005195 (PMC5147807; doi:10.1371/journal.pntd.0005195)
Supplement: S2 Table — (PDF) [file pntd.0005195.s006.pdf]

S2 Table. Detail of the villagers interviewed.

Village	Number of interviewees
Ban Donekang	21 villagers + the Head of Village (Nay Ban)
Ban Napho	19 villagers + the Head of Village (Nay Ban)
Ban Long Lao	19 villagers + the Head of Village (Nay Ban)
Ban Xieng Lome	19 villagers + the Head of Village (Nay Ban)
