# Supplementary material for: Hydrological Regime and Water Shortage as Drivers of the Seasonal Incidence of Diarrheal Diseases in a Tropical Montane Environment
Source: PLoS Negl Trop Dis. 2016 Dec 9;10(12):e0005195. doi: 10.1371/journal.pntd.0005195 (PMC5147807; doi:10.1371/journal.pntd.0005195)
Supplement: S3 Table — (PDF) [file pntd.0005195.s007.pdf]

S3 Table. Villagers interview matrix.

Social representations of vulnerability to water contaminations during floods and coping strategies.

Luang Prabang District, Laos.

Main objectives of the interview grid:

- Evaluate and create a hierarchy of perceived environmental risks inside the village and in the fields
- Collect qualitative information to determine how villagers feel vulnerable to the risk of water related disease, and which coping strategies adopted.

Themes/ Objectives	Questions
**<u>Occupation of the respondent</u>**<br><br>Objectives :<br>1) Evaluate the socio-economic level of the interviewee<br>2) Evaluate his he/she has a position in the village	- How old are you?<br>- How many children do you have? Do they still live in your house ? Do they work?<br>- How long have you been living in the village for? Are you a newcomer? Why did you move here ?<br>- Can you go easlely in Luang Prabang or in other villages ? In which villages do you go, and why? (family, work...). Which way of transport do you use ?<br>- Do you stand for a special position in the village ? If yes, why do you have this position? (age, wealth, gender, job...)
**<u>Water uses and practices inside and outside the village</u>**<br><br>Objectives:<br>1) Get indications on the possible moments and places of exposition to contaminations<br>2) Contextualize the feelings of vulnerability according to spatial criterion	- **<u>In the village</u>**<br><br>- Where are the important places in the village ?<br>- Are all the water good to drink here?<br>- What kind of water do you use, in the rainy season / In the dry season?<br>o *To drink*<br>o To *cook*<br>o *To wash (dishes, clothes…)*<br>o *To take a shower*<br>o *For your animals*<br>- Do you have water in your house (= nam lin) ? Since when ?<br>o Is it expensive ?<br>o Is it working well ?<br>- Is there a place where it is more dangerous to get sick because of water ?<br>- Do you go to the river sometimes ? What for? Is there somewhere where it's polluted ? How do you know it's polluted ?<br><br>- **<u>In the Fields</u>**<br><br>- What do you grow? why ? (subsitance/cash crops)<br>- Do you think that your surrounding environment has changed in the last years? Why ? Advantages ? Inconvenients ?<br>- Who comes and work with you when you are in the fieds ?<br>- What kind of difficulties do you face in your daily work?<br>- Are there any special risks regarding your farming activity? If yes,

	how do you cope with it?<br>- In the fields, what king of water do you use?<br>o For irrigation/Animals<br>o For Cooking<br>o For Drinking<br>- How do you make a difference between which water is good to drink and which is not ?
**<u>Water as an environmental risk</u>**<br><br>Objectives :<br>1) Evaluate the capacity of anticipation of risks: shortages or floods<br>2) Evaluate the feeling of vulnerability towards floods	- Is there water all year long?<br>- What do you do if there is a shortage? (cooking? Washing? Shower? Animals?)<br>- How do you protect yourself and your family?<br>- Are there floods sometimes?<br>- What do you do if there is a shortage? (cooking? Washing? Shower? Animals?)<br>- How do you protect yourself and your family?<br>- How big is the river when it's « normal » ? When it's dry ? Flooded ?<br>- What is the impact of floods on your fields ?
**<u>Water contaminations and health risks</u>**<br><br>Objectives:<br>1) Understand how water related diseases are perceived<br>2) Evaluate what are the health practices used to protect from important diseases	- Are the people in your family often sick ?<br>- How did you get cured ? (traditional healer, pharmacy, hospital?)<br>o Is it expensive? Are you sometimes reluctant to go there because of money ?<br>o Where can you buy medicines ?<br>o in the village?<br>o In the market?<br>o In Luang Prabang?<br>- Do you always go buy your medicine at the same place? Why?<br><br>- Why are you getting sick ? (mosquitoes, water, air, meat…)<br>- When are those diseases dangerous ?<br>- How can you protect yourself ?<br>- If someone is getting sick in the village, who can take care of him/her ?<br>- Is it easy to find people to help you in the village?<br>- Are you sometimes sick because of water?<br>o Which water? (cooking, cleaning, shower, drinking)…<br>o Where? (house/village/fields…)<br>o Which ^period of year?<br>- How can you protect yourself in order not to be sick because of water?<br>o What can you do when you are sick?
