# Supplementary material for: Hydrological Regime and Water Shortage as Drivers of the Seasonal Incidence of Diarrheal Diseases in a Tropical Montane Environment
Source: PLoS Negl Trop Dis. 2016 Dec 9;10(12):e0005195. doi: 10.1371/journal.pntd.0005195 (PMC5147807; doi:10.1371/journal.pntd.0005195)
Supplement: S5 Table — (PDF) [file pntd.0005195.s009.pdf]

S5 Table. Reported cases (i.e., hospital admissions) of diarrheal diseases from 2010 to 2012 across Luang Prabang area, Laos, by (1) the age of the patients, (2) their sex and (3) the spatial subset.

	**Age and sex group**	**% of the population**	**Reported cases**	**% of the reported cases**
**5 districts**	0-4	15	1668	50
	5-9		363	11
	10-14		217	6
	>14		1119	33
	Female	50	1534	46
	Male	50	1833	54
**Xieng Ngeun district**	0-4	17	66	62
	5-9		11	10
	10-14		9	8
	>14		20	19
	Female	50	33	31
	Male	50	73	69
**Houay Xon catchment**	0-4	11	162	50
	5-9		34	11
	10-14		17	5
	>14		108	34
	Female	48	129	40
	Male	52	192	60
**Houay Pano catchment**	0-4	14	7	32
	5-9		1	5
	10-14		2	9
	>14		12	55
	Female	52	11	50
	Male	48	11	50
