# Supplementary material for: Hydrological Regime and Water Shortage as Drivers of the Seasonal Incidence of Diarrheal Diseases in a Tropical Montane Environment
Source: PLoS Negl Trop Dis. 2016 Dec 9;10(12):e0005195. doi: 10.1371/journal.pntd.0005195 (PMC5147807; doi:10.1371/journal.pntd.0005195)
Supplement: S6 Table — The last 3 columns present the results of the LRT and show the significances of each variable, correcting for the potential confounding effects of the other variables. ar1 is the 1-step lagged incidence variable. (PDF) [file pntd.0005195.s010.pdf]

S6 Table. Effect of environmental variables on total incidence from May 2011 to December 2012. The last 3 columns present the results of the LRT and show the significances of each variable, correcting for the potential confounding effects of the other variables. ar1 is the 1-step lagged incidence variable.

	**Estimate**	**Std. Error**	**z value**	**Pr(>|z|)**	**Df**	**Deviance**	**Pr(>Chi)**
(Intercept)	0.5820	0.3425	1.6991	0.0893	-	-	-
ar1	0.0866	0.0176	4.9095	<0.0001	-	-	-
Rainfall_LP	-0.0065	0.0049	-1.3246	0.1853	1	1.7038	0.1918
Rainfall_HP	0.0064	0.0054	1.1927	0.2330	1	1.3700	0.2418
Air_Temp_HP	0.0049	0.0135	0.3638	0.7160	1	0.1306	0.7178
Discharge_S4	-0.0035	0.0026	-1.3243	0.1854	1	2.0972	0.1476
Discharge_NK20	-0.0004	0.0003	-1.3097	0.1903	1	2.3357	0.1264
